# A Dental Miscellany

**Published:** 1867-07

**Authors:** George L. Paine


### A DENTAL MISCELLANY.

BY GEORGE L. PAINE, D.D.S.

Read before the Mad River Valley Association.

Gentlemen:—We do not intend to perpetrate an address or essay, because we have not had the time nor health necessary to perform such duty. We shall give nothing but a sort of Dental hash, or *Olla Podrida*—a sort of Salmagundi, if you please.

One Sabbath morning, some months ago, just as we were about starting to church, a young man called and desired to be relieved of a tooth, stating that he had suffered severely during the preceding night, and must have relief if possible. We examined his teeth, and found them sound as a Mexican dollar—not a defect could we find. He pointed out the offending organ, which was a right superior cuspid, not a defect or sign of decay could we find—no sign of inflammation, though some soreness was manifest upon percussion. Having located the trouble, we decided to drill into the tooth, piercing the nerve, thus carrying the war into Africa. We started the drill in the crown, on a line parrallel with the axis of the tooth. When we had reached the nerve there was an escape of gas and some odor, which was the quintessence of fetor. Relief was at once afforded. We made an application of carbolic acid and directed him to call the next morning. The succeeding day we saw him, according to appointment, and he stated that he had slept splendidly the preceding night. We then applied White's preparation to the nerve, and the next day extracted the same. We then treated the canal with creosote and iodine, and filled it in a few days, all pain, soreness and tenderness having disappeared before filling; and at the end of two weeks he stated to me, as he was leaving for his far-off Western home, that said tooth was as useful and comfortable as any in his head. We only adduce this case as one of many similar ones which might be cited, to show the *folly* of *extracting teeth*, (as is too often done) which, with a little patience, study and perseverance, might be saved. This man desired to have his tooth extracted, but we positively refused to do so. He had no faith that it could be cured; but the result was, in a high degree, satisfactory to him. Daily, teeth are ruthlessly extracted which might and should be saved.

ANNEALING GOLD FOIL.—Our time, and the temper of plugging instruments, may be saved by annealing our foil before cutting the rope into pellets, by simply passing the gold rope through the flame of a spirit lamp.

When there is a copious flow of saliva in the mouth, it is often exceedingly difficult, if not sometimes impossible, to insert gold fi ling in an inferior molar and keep the gold and cavity perfectly dry. Moisture is fatal, and insures the failure of the plug. To secure absolute dryness, in filling under teeth, we have found nothing equal to Hawe's tongue-holder. It is just the thing. It beats the rubber dam or any other arrangement of which we have any knowledge.

When a cavity is tender, or the nerve nearly exposed, we like Hill's stopping, and often use it in such cases. We filled a tooth with this preparation five years ago, an approximal cavity, and it and the tooth are both good to this day. We never, on removing one of these fillings, found any fresh decay, or disintegration of the dentine, but found, on the contrary, all right and the cavity dry as Cromwell's powder.

Much pain sometimes follows the extraction of a tooth,—especially is this so when there is periostitis. This may be entirely removed in a few moments by saturating the alveolus with a mixture of creosote and iodine, or by the use of phenol sodique, a most invaluable remedy for wounds, cuts, etc., and an excellent hæmostatic.

In filling bicuspid teeth or molars, on their approximal surfaces, the cavity often extends to the edge of the grinding surface, with perhaps only the enamel intervening. Our plan, in such cases—in most cases, in fact, when proximal fillings are required in these teeth—is to chisel the cavity open from beneath, like a mortise, slightly dovetailing or grooving the same, and then fill from beneath. We can make a better filling, and *thus* prevent the breaking of the enamel, which is almost certain to occur sooner or later when these teeth are otherwise filled.

How often we—how much too often—all of us, are requested to extract teeth, just because they are aching. Why not lop off any other member—a finger for instance—because of a sore, or any kind of inflammation? Tooth-ache is as curable, in most cases, as any other disease. Then why extract so many teeth, so recklessly, so indiscriminately, as is so often the custom? Let us teach our patients the importance of saving their natural teeth—teach them to take good care of them—to cleanse them daily, thrice daily if need be, rather than have them lost through neglect. Let them be taught to bestow the same attention to their teeth that they do to their hair, their faces, their dress, and thousands of teeth will be saved from the remorseless fangs of the forceps, and fewer artificial teeth will be demanded.

The other day a gentleman visited us, who has been a patient of ours for many years, and whose teeth have always been hard, firm and of most excellent quality, but little inclined to decay, till within a recent period. For a few months he has been a victim to dyspepsia, and his teeth now bear witness that acid has been vigorously at work, making savage inroads upon those once beautiful organs. Now they are brittle, and look as if they have had a long bath in some kind of acid. I directed him to use antacids, hoping thereby to counteract, to some extent, the destructive tendency of the fluids of his mouth. Our people need to be taught how to eat—what to eat—in order to enjoy good health and long life, and especially good teeth.

We know a man, who, as soon as his first-born was provided with a pair of teeth, presented his wife with a miniature tooth-brush, with positive instructions that it be punctually used thrice, daily. What a noble example we have here! how worthy of imitation! how desirable that all parents should do likewise, and as urgently insist upon the regular use of the tooth-brush upon the teeth of all their children, and make it an imperative rule that as much attention shall be bestowed upon their teeth, to keep them perfectly clean, as their faces! The other day a lady brought her son to us, to have his teeth examined. We found a most beautiful set, symmetrically arranged, and perfectly clean. We spoke approvingly of the clean and healthy condition of his teeth and gums. Said she, "I brushed his teeth *daily* from the time he had two teeth until he was old enough to attend to that duty himself, and now that he has formed the habit, it is quite as natural for him to wash his teeth as it is to wash his face and hands. As children only know what they are taught, why not include this item of Dental hygiene in the programme of studies to which their minds are to be directed? Is not this knowledge quite as important to them as many other kinds for which, in many cases, they have no use in after life? Let us learn, and let us teach others to wash and be clean. Let this washing be not limited to the teeth, but extended so as to include the whole body. Dentists should see to it, that they obey this law—that their persons and clothing be strictly clean, then if they educate their conscience and keep that clean and bright, success must crown their professional efforts.

A matter of engrossing and widely spread interest, at this time, both among Dentists and the people, is Nitrous-Oxyd. Every one who has a sick tooth whose removal is fated, is anxious to take something to mitigate or prevent the pain of its extraction. Every Dentist now-a-days is expected to have a machine with which to make gas, (laughing-gas, we mean) else he is in danger of being ruled out of the ring, classed among the fossils. Well, since it is the fashion, and a necessity, to have a machine or apparatus, it is a matter of no small importance to have the *best* in the market. But just here is the rub: whose is the best? Every man thinks he has the best wife. We really don't know. It is a question we can't decide. We are disposed, however, to think that good gas can be made on any of the many machines in the market. We are not able to decide whose is the best, Leslie's, Mosely's, Barker's, or Sprague's. We should like to be satisfied on this subject, and hope some of our scientific men will determine, by actual tests, so that we may know where to invest, to the best advantage. Expense is a minor consideration, when a life may depend on the quality of the gas made.

Well, the pain of extracting teeth is, in some cases, and to many persons, exceedingly severe; hence it is very desirable that some safe and efficient anæsthetic agent should be kept and used to relieve suffering humanity. We believe that nitrous-oxyd is the safest and most reliable one in use. Prof. Barker says, "that nitrous oxide, or protoxide of nitrogen, is worthy to be placed prominently in the list of anæsthetics, is a fact which is daily being demonstrated by many of the most prominent surgical and Dental practitioners." Yet, it is not entirely safe in all cases. An agent that is so powerful—one that takes the patient so near the spirit land—should be used cautiously and with discrimination. We think that there are conditions of the system in which it is wholly unsafe to administer the gas—that the risk is too great to justify or warrant its use. Barker says: "Where there is a tendency to cerebral inflammation, disease of heart, either functional or organic, active congestion, or acute inflammation of the brain, lungs or kidneys, the use of this agent is contra-indicated." Says he, "I should use it with the greatest caution, where there is a general plethoric condition, or where there is a tendency to the hemorrhagic diathesis." "The danger of nitrous-oxide," says he, "consists in the fact that it increases the oxidation of the fluids and solids of the body, and also acts as a stimulant to the brain and nervous system. When there exists any predisposition to congestion or inflammation, the administration of nitrous-oxide may develope this latent tendency, and a fatal result may ensue." We suggest to our brethren that they do not rush in heedlessly, where angels fear to tread; and that they exercise discrimination and judgment in the use of this valuable, but potent agent.

To day we were thinking, indeed we have often had our thoughts run in the same vein, about cheap materials for filling teeth. We have long ago decided that there is no use in trying to stave off this subject of cheap fillings, or cheap materials for fillings There are some cases where it would be unprofitable, to both patient and practitioner, to use gold. All teeth are not, in all subjects, worth filling with gold—supposing it could be done. All persons are not able to pay for large and difficult gold fillings. What shall we use then? I have heard Dentists say they never fill teeth with anything but gold. Then there are some teeth that they don't fill, or if they do, their fillings don't last very long. Shall we use tin foil? This may do in approximal cavities, but it will not last in the grinding surface of a tooth; being soft, it wears away rapidly, requires too frequent renewals, and is, in the end, more expensive than gold. We like gold better than anything else. The conscience of a Dentist feels better after completing a good gold filling, and he can announce to his patient his fee with more complacency and a better grace than after perpetrating any other kind of filling. Other fillings than gold, anybody can insert, just as anybody, in three weeks, can learn to mount teeth on rubber; but to make good gold fillings, or mount artificial teeth on gold, demands science, skill and high art.

We, without any hesitation, know and teach and preach to all, that gold is always, and for everybody, with some few exceptions, the *healthiest*, the cheapest and the *best* material for filling teeth We do occasionally, insert and have inserted amalgam fillings, but *never* from choice, but *necessity*. We have, in our own teeth, three of the old-fashioned amalgam fillings, inserted twenty years ago, and they are good yet, and so are the teeth holding them. I honestly believe that, though modern Dental skill might save such teeth, there was not a Dentist in the West, twenty years ago, that could have filled our three teeth with gold so as to have saved them as perfectly as the amalgam has done. Teeth are filled now that twenty years ago would have been extracted without any remorse or hesitation. Artificial cusps, artificial halves, and in some instances, whole crowns of gold are forged out of gold foil, by the skill of the cunning Dentist, and thus made to do good and lasting service. Let us, therefore, be thankful to that master spirit who, through much ridicule, introduced to our profession, the knowledge and use of the mallet, by whose delicate and magic strokes such a revolution for good has been introduced in the art of filling teeth.

